# Workplace bullying, symptoms of anxiety and the interaction with leadership quality – a longitudinal study using dynamic panel models with fixed effects

**DOI:** 10.5271/sjweh.4060

**Published:** 2022-12-30

**Authors:** Rebecka Holmgren, Kathrine Sørensen, Louise Dalsager, Reiner Rugulies, Viveca Östberg, Linda L Magnusson Hanson

**Affiliations:** 1 Stress Research Institute, Department of Psychology, Stockholm University, Sweden; 2 National Research Centre for the Working Environment, Copenhagen, Denmark; 3 Department of Psychology, University of Copenhagen, Denmark; 4 Department of Public Health, University of Copenhagen, Denmark; 5 Department of Public Health Sciences, Stockholm University, Sweden

**Keywords:** harassment, mental health, occupational health, psychosocial work environment, structural equation modelling, work stress

## Abstract

**Objectives:**

Workplace bullying has been suggested to increase symptoms of anxiety. A reverse relationship has also been proposed. However, so far only few earlier studies have investigated this topic and the reported associations might partly be explained by unmeasured individual characteristics. In this study, we aim to examine the temporality and directionality between workplace bullying and anxiety symptoms, taking time-invariant characteristics into account. Furthermore, we aim to examine whether leadership quality modifies these associations.

**Methods:**

We included 13 491 individuals from two nationwide cohort studies in Sweden and Denmark. Using cross-lagged structural equation models (SEM) and dynamic panel models with fixed effects, we examined contemporaneous and lagged associations between self-reported workplace bullying and anxiety. Cohort-specific results were estimated and combined using fixed-effect meta-analysis.

**Results:**

The cross-lagged SEM models supported contemporaneous and lagged relationships in both directions (from workplace bullying to symptoms of anxiety and vice versa). In contrast, only contemporaneous relationships remained statistically significant and of considerable magnitude in the dynamic panel models with fixed effects. Specifically, exposure to workplace bullying was related to a concurrent increase in anxiety symptoms (b=0.61, 95% confidence interval 0.32–0.90). No support of interaction with leadership quality was found.

**Conclusions:**

The results indicate that onset of workplace bullying is associated with an immediate or short-term increase in anxiety symptoms. This study provides novel insights regarding temporal aspects and causal inference of the bullying-anxiety relationship useful for managing psychological hazards and preventing mental illness at work.

Workplace bullying is considered one of the most hazardous social stressors at work (1), with an estimated global prevalence of 11–18% (2). A common definition is to be repeatedly, and over a prolonged period, exposed to negative social acts at work with a perceived inability to defend oneself against these acts (3). These social acts are mainly of psychological nature and might include (but are not limited to) being socially excluded, being humiliated in front of others and being withheld important information (3).

There is evidence of a link between workplace bullying and onset of mental health problems, suggesting that workplace bullying can lead to increased symptoms and diagnosis of depression (4–6), increased suicidal ideation (7) and increased use of psychotropic medication (8). However, the prospective association between workplace bullying and anxiety has only received scarce attention (6, 9). To the best of our knowledge, this relationship has not yet been tested in a multi-wave study in the general working population, which would allow for an increased understanding of the association between change in exposure and outcome and thus of causality.

A reverse association has also been suggested (10). Not only may workplace bullying predict later symptoms of anxiety, but anxiety may in itself constitute a risk factor for later exposure to workplace bullying (11). The reverse association might result from workers with mental health problems ending up at unfavorable workplaces, mental health problems causing individuals to interpret their social surroundings negatively, or ill-health entailing less resources and hence vulnerability for being exposed (12).

This, potentially bidirectional, association might also partly be explained by time-stable individual characteristics, such as history of mental health problems and personality traits, which have been linked to increased risk of both workplace bullying and anxiety symptoms (13, 14). This imposes a methodological challenge, since these covariates might be difficult to measure and fully adjust for. Consequently, when comparing exposed to non-exposed individuals, the groups might not be interchangeable and therefore the differences that are found cannot confidently be derived from exposure status (15).

In order to further understand the influence of workplace bullying on mental health, moderating factors also need to be examined (16). Social support is recurrently theorized to buffer work-related stress (17) and has been found to moderate the relationship between workplace bullying and general mental distress (18). In particular, supervisory support seems to be of importance in understanding the effects of workplace bullying (18, 19). Supportive leadership, and other forms of leadership styles, have previously mainly been investigated as an antecedent for workplace bullying and these findings consistently indicate that leadership plays a crucial role (20, 21). This is in line with general leadership research, which repeatedly point at the beneficial effects that leadership styles, such as transformational (ie, engaging in motivation-enhancing behaviors) and supportive, have on employee health and well-being (22, 23). It thus seems reasonable to assume that perceived leadership quality, considered as supportive and transformational leadership behaviors, may buffer the relationship between workplace bullying and anxiety. To the best of our knowledge, this has not previously been examined.

The aim of this study is thus firstly to examine both the temporality and directionality between workplace bullying and anxiety symptoms, accounting for unmeasured time-stable characteristics, using a longitudinal three-wave design. Secondly, we aim to explore if leadership quality interacts with bullying and/or anxiety.

## Methods

### Study design and population

**Figure 1:**
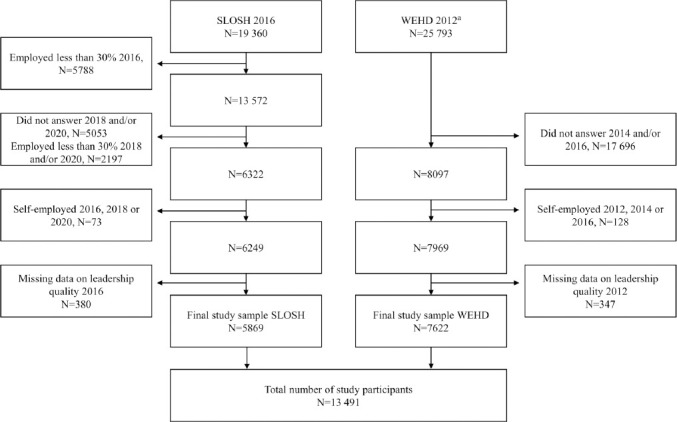
Flowchart describing selection of study participants. ^a^ All invited participants working ≥ 35 hours/ month.

The study population was derived from two Nordic cohort studies: the Swedish Longitudinal Occupational Survey of Health (SLOSH) and the Danish national questionnaire Work Environment and Health in Denmark (WEHD) study. Both SLOSH and WEHD are longitudinal surveys sent out biennially, focusing on associations between work environment and health (24, 25). SLOSH was initiated in 2006 and now includes 40 877 individuals, aged 16–64 at inclusion, approximately representative for the Swedish working population. The cohort is followed-up with two self-report questionnaires – one directed at individuals working ≥30% of full-time (as opposed to the one for individuals not working/working <30%) which was used for this study. WEHD was initiated in 2012 and the initial questionnaire was distributed to 34 805 individuals, aged 18–64 at inclusion, who were randomly sampled from the Danish working population (25). More detailed information regarding the cohorts and their respective response rate has been published elsewhere (24, 25) and can be found in the supplementary material (www.sjweh.fi/article/4060, text S1). Based on item availability, SLOSH questionnaires from 2016, 2018 and 2020 and WEHD questionnaires from 2012, 2014 and 2016 were used. Participants who had answered the questionnaires at all three time points, were not self-employed, and had provided information about leadership quality at time point 1 were included, as illustrated in figure 1. This resulted in a total study sample of 13 491 individuals, 5869 from SLOSH and 7622 from WEHD.

### Measurements

*Workplace bullying*. In SLOSH, workplace bullying was measured by asking participants to what extent they had “been subjected to personal persecution in the form of unkind words or behaviors from superiors or fellow workers” during the last six months. In WEHD, participants were provided with a formal definition of workplace bullying and were then asked if they had been exposed to bullying at work during the last 12 months. Answers were dichotomized (no/yes), using affirmative responses to indicate exposure. See supplementary material (text S2) for further details regarding measurements.

*Symptoms of anxiety*. Symptoms of anxiety were assessed by means of SCL-ANX4, a subscale of the Symptom Checklist-25 (26). Participants were asked to rate the frequency of a set of symptoms (see supplementary text S2) during the last week (in SLOSH) or during the last 4 weeks (in WEHD) on a 5-point scale. A sum score (range 0–16) was used in all analyses (27). For descriptive purposes, a score of ≥6 points was used to indicate clinically elevated anxiety symptoms (26). SCL-ANX4 has previously shown good psychometric properties (26). Cronbach’s alpha showed acceptable internal consistency (SLOSH time 1, α=0.78, WEHD time 1, α=0.77).

*Leadership quality*. In SLOSH, leadership quality was captured using the 10-item subscale leadership climate, from The Stress Profile (28), measuring the occurrence of specific leadership behaviors (28, 29) (see supplementary text S2). Items were rated on a 4-point scale. In WEHD, leadership quality was measured with 8 items, derived from leadership climate and the Copenhagen Psychosocial Questionnaire. Items were rated on a 5-point scale. Unidimensionality has been confirmed for this scale through factor analysis (30). Missing values were replaced with the mean if participants had answered ≥7 items (SLOSH) or ≥6 items (WEHD) and a sum score was calculated. Median split was used to define absence/presence of good leadership quality. Cronbach’s alpha showed high internal consistency (SLOSH time 1, α=0.89, WEHD time 1, α=0.86).

### Covariates

Covariates were chosen based on previous research and included sociodemographic information and job strain, the latter chosen as the sole indicator of poor psychosocial working environment to reduce model complexity. Sociodemographic covariates included registry-based sex (male/female), age (grouped into 18–<35/35–<45/45–<55/>55 years), educational attainment (considered low if having primary or secondary education, intermediate if having post-secondary education <3 years, and high if having post-secondary education >3 years or longer) as well as marital status (married/cohabiting or single). Job strain was measured at all time points by the combination of high demands and low decision authority (based on the sample median), using items from the subscales “demands” and “decision authority” from the Demand-Control-Support-Questionnaire (31) (see supplementary text S2). These covariates have previously been linked to both an increased risk of exposure to workplace bullying and an increased risk for symptoms of anxiety (14, 32–34).

### Statistical analysis

We fitted a series of structural equation models, analyzing contemporaneous and longitudinal associations separately. All models were adjusted for age, sex, marital status and educational attainment (time-invariant), as well as job strain (time-varying, modelled with autoregressive paths and paths to contemporaneous/lagged outcome). The first model included only autoregressive paths between exposure and outcome (M1: autoregressive). In addition, the following models included paths from exposure to outcome measured at the same time (M2: contemporaneous paths from bullying to anxiety and M3: contemporaneous paths from anxiety to bullying) and lagged paths from exposure to outcome (M4: lagged paths from bullying to anxiety, M5: lagged paths from anxiety to bullying). Lastly, we fitted a reciprocal model, with lagged paths from bullying to anxiety as well as lagged paths from anxiety to bullying (M6: reciprocal).

To account for both lagged dynamics and unmeasured time-invariant covariates, we carried out analyses with dynamic panel models with fixed effects (DPM), by means of structural equation modelling (see supplementary figure S1 for a graphical illustration). These models relies on the assumption of sequential exogeneity meaning that the exposure variables are assumed to be predetermined by their past values (35). Further, only variation within individuals are used for estimations, through inclusion of a latent variable (alpha, representing all time-stable individual characteristics) that is correlated with all time-varying predictor variables (35). In order to decrease model complexity, we fitted separate models for associations in different directions. The first (forward model) included change in workplace bullying as exposure and anxiety symptoms (both contemporaneous and lagged) as outcome. The second (reverse model) included change in anxiety symptoms as exposure and workplace bulling (both contemporaneous and lagged) as outcome. Possible reciprocal causation is still accommodated for by allowing the error term in each equation to correlate with future values of the predictor variable (36). Models were adjusted for job strain (time-varying). We estimated model parameters with diagonally weighted least squares (DWLS), using bootstrap to calculate robust standard errors. Missing values were handled by listwise deletion.

Model fit was evaluated by CFI, TLI, RMSEA and SRMR, using recommended values for good fit (<0.05 for RMSEA and SRMR and near 1 for CFI and TLI) (37). We considered SRMR as the most reliable fit measure, as the models included several categorical variables and some of them had small degrees of freedom (38). In addition, we used Chi-square difference tests to compare the SEM models.

Unstandardized regression coefficients (b) are presented, with 95% confidence intervals (CI). Allowing the regression coefficient of interest to vary at different time points did not significantly increase model fit, therefore constrained regression coefficients (set to be equal at all time points) were used.

We examined the interaction with leadership quality by adding perceived leadership quality at time point 1 to the dynamic panel models as a time-invariant variable, together with an interaction term between leadership quality and the predetermined exposure variable.

We performed four sensitivity analyses based on DPM models: (i) main analyses performed on full sample using full information maximum likelihood; (ii) restricting the sample to only include participants who had stayed within the same employer during all three waves (only possible for SLOSH, N=4384); (iii) interaction analysis using 4 cohort-identical items only (see supplementary text S2); and (iv) main analysis stratified by sex.

Lastly, we combined all cohort specific estimates in a meta-analysis, using fixed effects approach to account for variations in measurements between SLOSH and WEHD. Heterogeneity of the results was tested for using I^2^ statistics.

STATA version 16 (Stata Corp, College Station TX, USA) and R Studio version 1.4.1717 (packages lavaan, dpm and metafor) were used.

## Results

The two samples together amounted to 13 491 employed individuals. The majority of the included individuals were female, aged ≥45, and married or cohabiting (see table 1). About half of the individuals reported some post-secondary education and 16.6% held leadership responsibilities. The group exposed to workplace bullying included a slightly higher proportion of females, singles and individuals with leadership responsibilities and had lower educational attainment (data not shown).

**Table 1:** Descriptive statistics of study participants ^a,b^. [SLOSH=Swedish Longitudinal Occupational Survey of Health; WEHD=Work Environment and Health in Denmark; SD= standard deviation.]

	SLOSH N=5869		WEHD N=7622		Total N=13491	
		
	% (N)	Mean (SD)	% (N)	Mean (SD)	% (N)	Mean (SD)
Sex						
Female	60.0 (3524)		55.2 (4210)		57.3 (7734)	
Male	40.0 (2345)		44.8 (3412)		42.7 (5757)	
Age (years)						
<35	4.7 (237)		12.3 (936)		8.7 (1173)	
35–<45	19.4 (1139)		23.4 (1780)		21.6 (2919)	
45–<55	39.8 (2338)		37.8 (2880)		38.7 (5218)	
>55	36.1 (2119)		26.6 (2026)		30.7 (4145)	
Marital status						
Married/cohabiting	79.3 (4615)		80.3 (6121)		79.9 (10736)	
Single	20.7 (1205)		19.7 (1501)		20.1 (2706)	
Educational attainment						
Low	40.3 (2366)		53.9 (4073)		47.9 (6439)	
Intermediate	7.9 (464)		32.8 (2477)		21.9 (2941)	
High	51.8 (3036)		13.4 (1013)		30.2 (4049)	
In leadership position						
Yes	33.0 (1882)		4.2 (325)		16.6 (2207)	
No	67.0 (3813)		95.7 (7244)		83.4 (11057)	
Exposure workplace bullying						
Yes	7.9 (462)		12.2 (925)		10.3 (1387)	
No	92.1 (5386)		87.8 (6660)		89.7 (12046)	
Anxiety		2.0 (2.5)		1.7 (2.5)		1.8 (2.5)
Yes	9.9 (576)		4.6 (349)		6.8 (13443)	
No	90.1 (5245)		95.4 (7273)		93.1 (12518)	
High leadership quality						
Yes	46.5 (2730)		44.2 (3369)		45.2 (6099)	
No	53.5 (3139)		55.8 (4253)		54.8 (7392)	
Job strain						
Yes	10.7 (623)		12.8 (975)		11.9 (1598)	
No	89.3 (5193)		87.2 (6616)		11809 (88.1)	

^a^ Missing data in SLOSH: % (N) sex: complete, age: complete, marital status: 0.8 (49), educational attainment: 0.1 (3), leadership position: 3.0 (174), workplace bullying: 0.4 (21), anxiety: 0.9 (48), leadership quality: complete, job strain 0.9 (53). Missing data in WEHD: % (N) sex: complete, age: complete, marital status: complete, educational attainment: 0.01 (59), leadership position: <0.01 (53), workplace bullying: <0.01 (37), anxiety: complete, leadership quality: complete, job strain: <0.01 (31).

^b^ All items measured at year of first wave included in study (SLOSH: 2016, WEHD: 2012).

At different time points, prevalence of exposure to workplace bullying varied between 8–9% in SLOSH and 11–12% in WEHD. Elevated anxiety levels were more commonly reported in the exposed group, compared to the non-exposed. Exposed individuals also perceived a lower degree of good leadership quality, and reported a higher degree of job strain, see supplementary material (table S1).

### Cross-lagged SEM

In both cohorts, the reciprocal model showed best fit compared to the less complex models, see supplementary material (table S2). Overall, the results from the cross-lagged SEM analyses (adjusted for sex, age, marital status, educational attainment and job strain) showed that exposure to workplace bullying was statistically significantly associated with contemporaneous anxiety symptoms and anxiety symptoms at later time points. In addition, paths from anxiety symptoms to workplace bullying were statistically significant, both when assessing contemporaneous and longitudinal relationships. Pooled regression coefficients together with 95% CI are presented in figure 2a-c. See supplementary material (tables S3 and S4>) for cohort-specific estimates. Considerable heterogeneity was found across the cohorts (I^2^ varying between 89 and 98%).

**Figure 2a:**
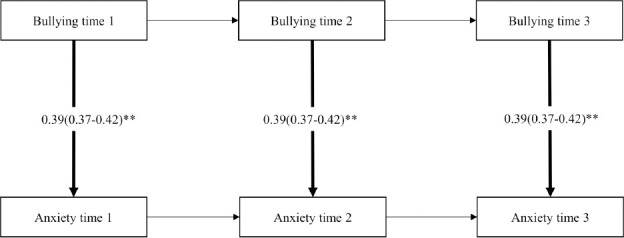
Pooled regression coefficients (b) and 95% confidence interval from SEM-model M2 (contemporaneous paths from bullying to anxiety). N=11 831. Model adjusted for sex, age, educational attainment, marital status (time-stable) and job strain (time-varying).

**Figure 2b:**
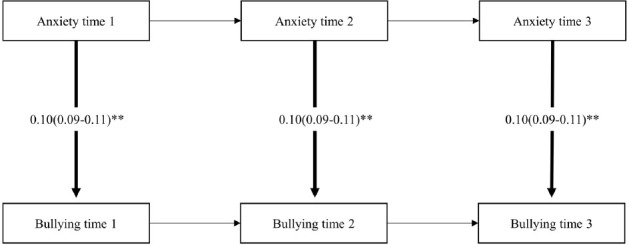
Pooled regression coefficients (b) and 95% confidence interval from SEM-model M3 (Contemporaneous paths from anxiety to bullying). N=11,831. Model adjusted for sex, age, educational attainment, marital status (time-stable) and job strain (time-varying). **P<0.001

**Figure 2c:**
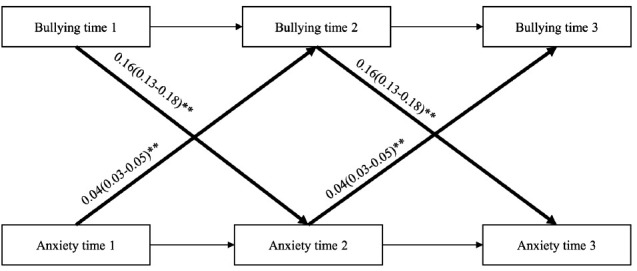
Pooled regression coefficients (b) and 95% confidence interval from SEM-model M6 (lagged reciprocal paths between bullying and anxiety). N=11,831. Model adjusted for sex, age, educational attainment, marital status (time-stable) and job strain (time-varying). **P<0.001

### Dynamic panel models with fixed effects

Becoming exposed to workplace bullying was associated with a contemporaneous increase (of 0.61 points, 95% CI 0.32–0.90) in anxiety symptoms, compared to levels of anxiety before exposure (see figure 3a). However, no change in symptoms across the later time points (“lagged effects”) was observed. In the reverse model, (using change in anxiety symptoms as exposure and workplace bullying as outcome) contemporaneous associations were not observed. However, a small lagged effect (b=-0.01, 95% CI -0.01–0.00) was observed (see figure 3b). See supplementary material (tables S3 and S4) for cohort-specific estimates. No heterogeneity was observed (I^2^=0%). Models showed good model fit (see table 2). Results from models without job strain were similar (data not shown).

**Figure 3a:**
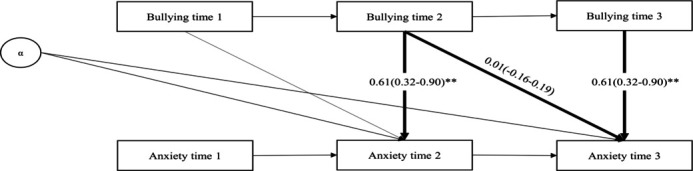
Pooled regression coefficients (b) and 95% confidence interval from DPM-model forward (contemporaneous and lagged paths from bullying to anxiety). N=11,986. Model adjusted for job strain (predetermined) and alpha (α, latent variable representing all time-stable characteristics). **P<0.001

**Figure 3b:**
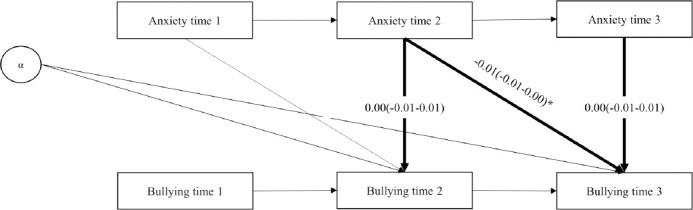
Pooled regression coefficients (b) and 95% confidence interval from DPM-model reverse (contemporaneous and lagged paths from anxiety to bullying). N=11,986. Model adjusted for job strain (predetermined) and alpha (α, latent variable representing all time-stable characteristics). *P<0.05

**Table 2:** Results from model fit indices for dynamic panel models with fixed effects^a^ performed in the Swedish Longitudinal Occupational Survey of Health (SLOSH) (N=5869) and Work Environment and Health in Denmark (WEHD) (N=7622) respectively. [CFI=Comparative Fit Index; TLI=Tucker-Lewis Index; RMSEA=root mean square error of approximation; SRMR=standardized root mean square residual.]

	df	N used	Chi-2	CFI	TLI	RMSEA	SRMR
Workplace bullying to anxiety (forward)							
SLOSH	1	5468	23.628	0.997	0.910	0.064	0.012
WEHD	1	6518	0.536	1.000	1.001	0.000	0.002
Anxiety to workplace bullying (reverse)							
SLOSH	1	5468	37.852	0.995	0.854	0.082	0.017
WEHD	1	6518	3.142	1.000	0.994	0.018	0.005

a All models adjusted for job strain (predetermined) and including alpha (latent variable representing all time-stable characteristics).

Lastly, we added interaction terms with leadership quality in both DPM models, but no statistically significant interaction was observed (pooled results from forward model: P=0.36, reverse model: P=0.46).

### Additional analyses

Sensitivity analysis using full information maximum likelihood to allow for inclusion of missing values yielded similar results (except that no lagged effects were found in the reverse model), as did restricting the sample to those who had stayed within the same employer between all timepoints (only performed in SLOSH) and stratifying the analyses by sex, see supplementary material (table S5). Using the shorter leadership quality scale did not result in any statistically significant interaction.

Attrition analysis (comparing the final study samples in each cohort to the excluded subjects who were gainfully employed at time 1) showed no differences regarding exposure to workplace bullying. Slightly higher levels of anxiety symptoms were reported among the excluded subjects compared to the final study sample. See supplementary material (table S6) for detailed results.

## Discussion

When using traditional SEM analysis, we found support for a bidirectional contemporaneous and longitudinal association between workplace bullying and symptoms of anxiety. However, when applying DPM, which by design also accounts for unmeasured time-invariant covariates, we observed a different temporal and directional pattern. Whereas exposure to workplace bullying was still related to a contemporaneous increase in anxiety symptoms, we did not find support for a time-lagged relationship with symptoms of anxiety. The results did not provide evidence of any reverse contemporaneous association, though, a small lagged reverse association was seen. Furthermore, we did not find any support for leadership quality modifying the explored associations.

Our results are in line with previous cross-sectional research reporting an association between exposure to workplace bullying and anxiety symptoms (11). In addition, we showed that, although attenuated, this association persists over and above what can be explained by time-stable individual characteristics, thus supporting the interpretation that there is a causal association between workplace bullying and anxiety symptoms. It should be acknowledged that our effect estimates are of small magnitude, as it is often observed in research on psychosocial job stressors (39).

In contrast to both previous research and our own results using a cross-lagged SEM models, we did not observe a lagged association between workplace bullying and anxiety symptoms two years later. Using different time lags (six months and five years respectively), both Rodriguez-Munoz and Moreno-Jimenez (9) and Einarsen and Nielsen (6) found that exposure to workplace bullying was associated with subsequent anxiety symptoms in nationally representative samples of the workforce. In addition, using a sample of Norwegian nurses, Reknes and Pallesen (40), found that workplace bullying was associated with increased levels of anxiety one year later. All of these studies however relied on the behavioral measurement method of workplace bullying (rating the frequency of specified negative acts, as opposed to the self-labelling method used in this study), which might have affected the explored associations (41). Einarsen and Nielsen (6) also examined self-labelled exposure to workplace bullying, with no significant longitudinal associations with anxiety. In our study longitudinal associations were found in the traditional SEM analyses only, but not in the DPM analyses. It is important to point out that the latter analytical approach allowed us to study the influence of change in exposure status, not the influence of prolonged exposure that may have been captured in previous work. Although more studies (using different time lags and applying the behavioral measurement method) are warranted, one possible interpretation of our results is that a long-term association with anxiety following exposure to workplace bullying might be overestimated because of time-invariant confounding. As far as we know, our study is the first to account for both measured and unmeasured time-invariant confounding in relation to workplace bullying. However, differences in specifications between our SEM and DPM models could also have contributed to the differences in results. Our results may also be explained by a focus on anxiety symptoms, which may be a more immediate indicator of stress/strain, rather than anxiety disorders. According to the allostatic load model eg, symptoms of anxiety and mood changes may be among the primary effects of an “allostatic load” (“wear and tear on the body”) resulting from exposure to stressors, while tertiary outcomes such as mental disorders are assumed to emerge only as a result of chronic stress dysregulation of bodily organ systems (42).

Reverse associations (symptoms of anxiety predicting onset of workplace bullying) were generally not observed in our study, with the exception of contemporaneous reverse associations in the traditional SEM analysis. The association did not remain statistically significant in the DPM model, which to a higher degree can separate cause from effect. In the reversed DPM model, a lagged association was found, in opposite direction than expected (increase in anxiety predicting lower risk of workplace bullying). This result needs to be interpreted with caution as the point estimate was very small (and was not found in the sensitivity analyses). It has also been suggested that the method used carries a risk of producing “artefactually negative results” if the time lag is misspecified (43).

We did not find support for any interaction between leadership quality and the main variables. A perceived good leadership quality did not seem to buffer the immediate or short-term increase in anxiety among those exposed to workplace bullying. Previous studies have suggested leadership to be an important moderator of work-related outcomes after exposure to bullying (44), however our results align with other studies failing to identify a buffering effect of leadership on the association between adverse working conditions and risk of psychological health problems (19). Although leadership quality is important in order to prevent the occurrence of workplace bullying (20), our results suggests that it might not be enough to reduce the risk for anxiety symptoms attributed to workplace bullying. In our study, we used a relatively broad concept of good leadership quality, encompassing both supportive and transformational leadership behaviors. It is possible that a narrower definition would have generated other results.

Stratifying our results by sex did not point towards any differences between how men and women were affected by workplace bullying. Similar studies have found other tendencies, with workplace bullying leading to increased anxiety in men but not in women (6). Further studies are needed to clarify the role of sex in mental health-related outcomes of workplace bullying.

### Strengths and limitations

A major strength of this study is the applied longitudinal design, using repeated measurements allowing us to study change in exposure. We had a relatively large sample size stemming from two independent cohorts, and our sample included sufficient individual change in exposure status, which enabled us to perform DPM modelling alongside more traditional SEM analysis. We chose statistical methods with consideration to the potential influence of time-stable individual characteristics as well and the possibility of reversed causality. The DPM models should, by design, attenuate these potential sources of bias (35). However, even though several time-varying covariates were included in both statistical models, we were not able to rule out the influence of unmeasured time-varying confounding. The impact of unmeasured time-varying covariates related to the workplace (eg, employment form and/or job insecurity) was on the other hand to some extent limited in our sensitivity analysis on a subsample that did not change employer between waves, and these analyses yielded similar results.

Several limitations should be acknowledged. Although repeated measures are highlighted as a general strength of the study, they might also introduce selection bias, as we restricted participation to those who were gainfully employed and had answered the questionnaire at all three time points. Prior findings indicate that repeated participation in SLOSH is more common among females, native-borns and higher educated individuals (24). Our own attrition analyses confirmed these patterns and also found a higher attrition rate among participants with increased anxiety levels, in both cohorts. This points towards a potential underestimation of the explored relationships and limit the generalizability of findings. However, even if the analyses were carried out among selected subjects, exposure–outcome relationships do not necessarily differ considerably due to non-response. Reported levels of anxiety was fairly low among our sample, and leadership ratings were generally high, further indicating a potential healthy worker effect (12). Furthermore, this study was carried out in a Scandinavian setting, were prevalence levels of workplace bullying are generally lower than in other parts of the world (2). Overall, this points to the importance of cautiousness when generalizing the results to other populations. Further, we chose a time lag of two years; however, it is possible that a shorter or longer time lag would have resulted in different estimates.

We used data from two separate cohorts, carried out in neighboring countries, in order to strengthen the robustness of the study. Although efforts were made to use as similar measurements as possible, there remains differences in how covariates were measured. The measurement of workplace bullying in SLOSH might be narrower than both the standard definition of workplace bullying and the measurement used in WEHD, potentially affecting the results. Sampling processes and timings of data collection also differed between the cohorts. This heterogeneity could not be fully considered in the meta-analyses. Therefore, we displayed both cohort-specific and pooled estimates, when possible.

Information on all study variables was gathered through self-reported questionnaires and might thus be sensitive to common method bias, although using data collected at different time points and DPM analysis to some extent can reduce this bias. We chose to measure leadership quality at time point 1 only, to avoid that the measurement was affected by potential exposure to workplace bullying by participants’ supervisors. However, we cannot be certain that the participants did not change supervisor between different time points. results.

Lastly, the DPM models all displayed good model fit. Results from model fit indices might, however, partially have been affected by the small degrees of freedom of the models. Less complex models and/or a greater number of observations could have increased the degrees of freedom.

### Concluding remarks

In contrast to previous findings, our results indicate that workplace bullying increased the risk of symptoms of anxiety in an immediate or short-term time frame, but not in the longer term, when using a lag of two years and accounting for time-stable individual characteristics. The results provide novel insights about the temporal aspects of the bullying-anxiety relationship and point towards primary prevention of workplace bullying as an important strategy for limiting proximal symptoms of anxiety. Leadership quality does not appear to modify these associations, which do not suggest that improvement in leadership is a promising alternative primary prevention strategy. Future studies, applying similar analytic strategies with shorter time lags are, however, warranted to clarify the longitudinal effects of workplace bullying on anxiety.

## Supplementary material

Supplementary material

## Protection of research participants

SLOSH has obtained ethical approval from the Regional Ethical Review Board in Stockholm [#2012/373‐31/5, #2006/158‐31, #2008/240‐32, #2008/1808‐32, #2010/0145‐32, #2012/373‐31/5, #2013/2173‐32, #20152187, #2015/2298‐32, #2017/25‐35‐32]. Questionnaire- and register-based studies do not require ethical approval in Denmark, WEHD was conducted according to the Helsinki Declaration (as revised in 2008) and has been registered by the Danish Data Protection Agency. All participants were given written information about the purpose of each study, and informed consent was obtained for each survey year.
