# Supplementary material for: Workplace bullying, symptoms of anxiety and the interaction with leadership quality – a longitudinal study using dynamic panel models with fixed effects
Source: Scand J Work Environ Health. 2022 Dec 30;49(1):64–74. doi: 10.5271/sjweh.4060 (PMC10549915; doi:10.5271/sjweh.4060)
Supplement: Supplementary material [file SJWEH-49-64-S001.pdf]

Workplace bullying, symptoms of anxiety and the interaction with leadership quality – a longitudinal study using dynamic panel models with fixed effects[1]

by Rebecka Holmgren, MSc,[2] Kathrine Sørensen, MSc, Louise Dalsager, PhD, Reiner Rugulies, PhD, Viveca Östberg, PhD, Linda L Magnusson Hanson, PhD

1. Supplementary material
2. Correspondence to: Rebecka Holmgren, Stress Research Institute at Department of Psychology, Stockholm University, 106 91 Stockholm, Sweden. [E-mail: rebecka.holmgren@su.se]

Supplementary text S1: Information about the cohorts

Swedish Longitudinal Occupational Survey of Health (SLOSH)

SLOSH is a Swedish longitudinal survey, focusing on work environment and health. Participants were initially sampled from the Swedish Work Environment Survey (SWES) 2003, which consists of a representative sample of the Swedish working population in that year. Follow-up questionnaires have been sent out biennially since 2006. The response rate in SLOSH 2006 was 65%. New participants were added to SLOSH from SWES in 2008, 2010 and 2014.

SLOSH questionnaires are sent out in two versions for participants to choose from: one for individuals working at least 30% (during the past three months) and one for individuals working less than 30% or who for some reason are not working (e.g. unemployed, student, retired). In total, 28 672 participants have responded to at least one of the two versions of the SLOSH questionnaire.

Reference: Magnusson Hanson LL, Leineweber C, Persson V, Hyde M, Theorell T, Westerlund H. Cohort Profile: The Swedish Longitudinal Occupational Survey of Health (SLOSH). Int J Epidemiol. 2018;47(3):691-2i.

Work Environment and Health in Denmark (WEHD)

WEHD is a biennial longitudinal survey, initiated in 2012, intended to study work environment and health among Danish employees. Participants were randomly sampled from all employed individuals (between 30 November 2011 and 31 January 2012), working ≥35 hours/month, aged 18 to 64 and living in Denmark. The initial questionnaire was distributed to 34 805 individuals, and had a response rate of 50.5%.

Reference: Johnsen NF, Thomsen BL, Hansen JV, Christensen BS, Rugulies R, Schlünssen V. Job type and other socio-demographic factors associated with participation in a national, cross-sectional study of Danish employees. Bmj Open. 2019;9(8).

Supplementary text S2: Information regarding measurements

Workplace bullying

SLOSH:

During the last 6 months, have you been subjected to personal persecution in the form of unkind words or behaviors from your superiors or fellow workers?

Response alternatives: Yes, weekly/Yes, monthly/Yes, sometime/No

WEHD:

Have you been exposed to bullying at work **within the past 12 months**? Bullying occurs when one or more individuals regularly and over a longer period of time – or repeatedly in a rough way – subjects one or more individual to abusive acts, which are perceived as hurtful or degrading by the targeted individual(s)

Response alternatives: Yes, daily/Yes, weekly/Yes, monthly/Yes, seldomly/No, never

If yes, who exposed you to bullying?*

Response alternatives: Colleagues/Supervisors/Subordinates/Customers, clients, patients, students

**To increase comparability between the cohorts, only respondents reporting having being exposed to bullying by colleagues, supervisors and/or subordinates were classified as being exposed to bullying at work.*

Symptoms of anxiety (SCL-ANX4)

SLOSH:

During the last week, how much were you bothered by:

1. Feeling suddenly scared for no reason?
2. Nervousness or shakiness inside?
3. Spells of terror or panic?
4. You worry too much?

Response alternatives: Not at all/Little/Moderately/Rather intensively/Intensively

WEHD:

During the last 4 weeks, how much were you bothered by

1. Feeling suddenly scared for no reason?
2. Nervousness or shakiness inside?
3. Spells of terror or panic?
4. You worry too much

Response alternatives: Not at all/Little/Moderately/Rather intensively/Intensively

Leadership quality

SLOSH:

1. My boss gives me the information I need

2. My boss is good at pushing through and carrying out changes
3. My boss explains goals and subgoals for our work so that I understand what they mean for my particular part of the task
4. I have a clear picture of what my boss expects of me
5. My boss shows that he/she cares how things are for me and how I feel
6. I have sufficient power in relation to my responsibilities
7. My boss takes the time to become involved in his/her employees' professional development
8. My boss encourages my participation in the scheduling of my work
9. I am praised by my boss if I have done something good
10. I am criticized by my boss if I have done something that is not good

Response alternatives: No, never/No, seldom/Yes, sometimes/Yes, often

Item 3, 6, 7 and 8 used to create 4-item scale used in sensitivity analysis.

WEHD:

1. Does your immediate manager explain the company's objectives so you understand that they mean for your work tasks?
2. Do you have sufficient authority in relation to your responsibilities?
3. Does your immediate manager take the time to engage in your professional development?
4. Does your immediate manager involve you in the planning of your work?
5. Does your immediate manager give you the necessary feedback (praise and criticism) for your work?
6. Is your work recognized and appreciated by the management?
7. Do you get the necessary help and support from the management?
8. Can you trust the information that comes from the management?

Response alternatives: Always/Often/Sometimes/Seldom/Never/I don't have a manager** ***item excluded from analysis if this alternative was chosen*

Item 1, 2, 3 and 4 used to create 4-item scale used in sensitivity analysis.

Job strain

An indicator variable for job strain was created through the combination of high demands and low decision authority, according to median split.

SLOSH:

Demands:
Do you have to work fast?
Do you have enough time to do everything?
Does your work often involve conflicting demands?

Decision authority:
Do you have a choice in deciding how you do your work?
Do you have a choice in deciding what you do at work?

Response alternatives: Yes, often/Yes, sometimes/No, seldom/No, never

WEHD:

Demands:
Is it necessary to keep a high work pace?
Do you have enough time for your work tasks?

Do you experience conflicting demands at your job?

Decision authority:
Can you influence when you solve your work tasks?
Can you influence how you solve your work tasks?

Response alternatives: Always/Often/Sometimes/Seldom/Never

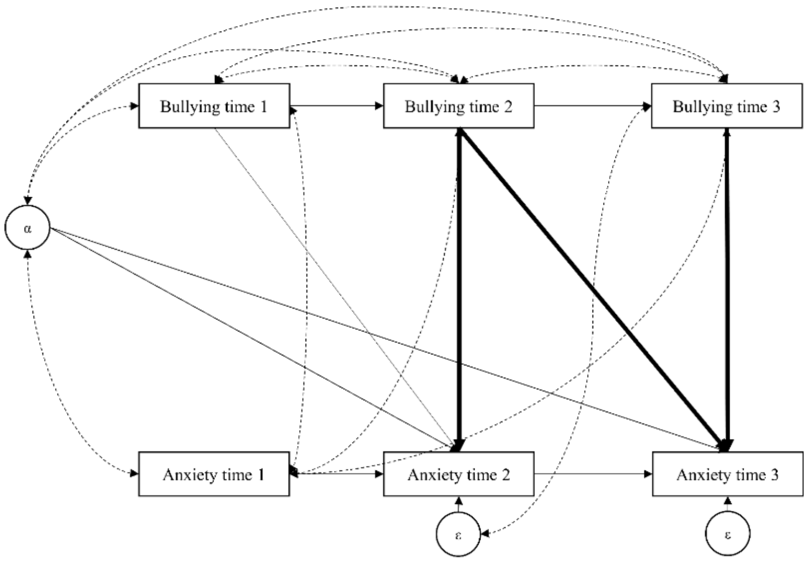

Supplementary figure S1.
Illustration of the crude dynamic panel model with fixed effects, using workplace bullying as exposure and symptoms of anxiety as outcome. Final models also included job strain (time-varying).

α = alpha, latent variable representing all time-stable confounders. ε = error term. Arrows indicating regression paths, bold arrows indicating contemporaneous and lagged associations of interest for study, dashed double-headed arrows indicating correlations.

Illustration based on figure 1 presented in following article:

Allison PD, Williams R, Moral-Benito E. Maximum Likelihood for Cross-lagged Panel Models with Fixed Effects. Socius: Sociological Research for a Dynamic World. 2017;3:1-17. doi: 10.1177/2378023117710578

Supplementary table S1. Main variables by exposure status[a].

	Total<br>N=13491<br>% (N)	Exposed<br>N=1387<br>% (N)	Non-exposed<br>N=12046<br>% (N)
**Anxiety**			
Yes	6.8 (13443)	*15.9 (219)*[b]	*5.9 (703)*
No	93.1 (12518)	*84.1 (1161)*	*94.1 (11303)*
**High leadership quality**			
Yes	45.2 (6099)	*21.6 (300)*	*47.9 (5776)*
No	54.8 (7392)	*78.4 (1087)*	*52.1 (6270)*
**Job strain**			
Yes	11.9 (1598)	*25.9 (358)*	*10.3 (1236)*
No	11809 (88.1)	*74.1 (1022)*	*89.7 (10752)*

[a] All items measured at year of first wave included in study (SLOSH: 2016, WEHD: 2012).
[b] Italics indicating significant differences between exposed and unexposed according to chi-square test, at p<0.05-level.

Supplementary table S2. Results from model fit indices for structural equation models[a] performed in SLOSH and WEHD respectively.

	df	N used	Chi-2	Chi-2 robust	CFI	TLI	RMSEA	SRMR	Model comparison			
**SLOSH (N=5869)**										**Δdf**	**Δχ2**	**p**
M1: Autoregressive	66	5373	549.049	727.922	0.909	0.950	0.043	0.134				
M2: B[b]→A[c]	73	5373	1131.070	1311.135	0.830	0.916	0.056	0.172				
M3: A→B	73	5373	1497.282	1667.804	0.781	0.892	0.064	0.156				
M4: B→A, lagged	64	5373	543.028	722.892	0.909	0.949	0.044	0.135				
M5: A→B, lagged	64	5373	511.453	687.473	0.914	0.952	0.043	0.124				
M6: Reciprocal, lagged	60	5373	426.198	578.242	0.929	0.957	0.040	0.107	*vs 1*	6	137.65	<0.001
									vs 2	13	465.84	<0.001
									vs 3	13	718.94	<0.001
									vs 4	4	125.43	<0.001
									vs 5	4	102.38	<0.001
**WEHD (N=7622)**												
M1: Autoregressive	66	6458	778.248	995.690	0.928	0.961	0.047	0.137				
M2: B→A	73	6458	1764.240	1974.683	0.854	0.928	0.064	0.173				

M3: A→B	73	6458	18741.290	13026.530	0.812	0.907	0.072	0.152				
M4: B→A, lagged	64	6458	759.588	971.071	0.930	0.961	0.047	0.137				
M5: A→B, lagged	64	6458	706.734	914.748	0.935	0.963	0.045	0.122				
M6: Reciprocal, lagged	60	6458	564.952	739.015	0.948	0.969	0.042	0.107	*vs 1*	6	237.61	<0.001
									vs 2	13	813.85	<0.001
									vs 3	13	1204	<0.001
									vs 4	4	201.48	<0.001
									vs 5	4	164.5	<0.001

[SLOSH=Swedish Longitudinal Occupational Survey of Health, WEHD=Work Environment and Health in Denmark. CFI=Comparative Fit Index. TLI=Tucker-Lewis Index. RMSEA=Root Mean Square Error of Approximation. SRMR=Standardized Root Mean Square Residual]

[a] All models adjusted for sex, age, educational attainment, marital status (time-stable) and job strain (time-varying).
[b] B=Workplace bullying
[c] A=Symptoms of anxiety

Supplementary table S3. Regression coefficients (b) from models using symptoms of anxiety as outcome. [SEM=Structural equation model, DPM=dynamic panel model with fixed effects, SLOSH=Swedish Longitudinal Occupational Survey of Health, WEHD=Work Environment and Health in Denmark, CI=Confidence interval.]

**Workplace bullying to symptoms of anxiety**				**Contemporaneous effects**		**Lagged effects**	
			**N**	**b**	**95% CI**	**b**	**95% CI**
**SLOSH**	**SEM**[a]	M2	5373	0.33**	0.29-0.38		
		M6	5373			0.07*	0.01-0.12
	**DPM**[b]	Forward	5468	0.56*	0.12-0.99	-0.04	-0.33-0.24
**WEHD**	**SEM**[a]	M2	6458	0.42**	0.39-0.45		
		M6	6458			0.19**	0.15-0.22
	**DPM**[b]	Forward	6518	0.66**	0.27-1.05	0.05	-0.18-0.27
**Total**	**SEM**[a]	M2	11831	0.39**	0.37-0.42		
		M6	11831			0.16**	0.13-0.18
	**DPM**[b]	Forward	11986	0.61**	0.32-0.90	0.01	-0.16-0.19

*p<0.05 **p<0.001.
[a] Adjusted for sex, age, educational attainment, marital status (time-stable) and job strain (time-varying).
[b] Adjusted for job strain (predetermined) and alpha (latent variable representing all time-stable characteristics).

Supplementary table S4. Regression coefficients (b) from models using exposure to workplace bullying as outcome. [SEM=Structural equation model, DPM=dynamic panel model with fixed effects, SLOSH=Swedish Longitudinal Occupational Survey of Health, WEHD=Work Environment and Health in Denmark, CI=Confidence interval.]
*p<0.05 **p<0.001.

**Symptoms of anxiety to workplace bullying**				**Contemporaneous effects**		**Lagged effects**	
			**N**	**b**	**95% CI**	**b**	**95% CI**
**SLOSH**	**SEM**[a]	M3	5373	0.07**	0.06-0.08		
		M6	5373			0.03**	0.02-0.04
	**DPM**[b]	Reverse	5468	0.00	-0.01-0.01	-0.00	-0.01-0.00
**WEHD**	**SEM**[a]	M3	6458	0.12**	0.11-0.13		
		M6	6458			0.05**	0.04-0.06
	**DPM**[b]	Reverse	6518	0.00	-0.02-0.02	-0.01	-0.02-0.00
**Total**	**SEM**[a]	M3	11831	0.10**	0.09-0.11		
		M6	11831			0.04**	0.03-0.05
	**DPM**[b]	Reverse	11986	0.00	-0.01-0.01	-0.01*	-0.01-0.00

[a] Adjusted for sex, age, educational attainment, marital status (time-stable) and job strain (time-varying).
[b] Adjusted for job strain (predetermined) and alpha (latent variable representing all time-stable characteristics).

Supplementary table S5. Regression coefficients (b) from dynamic panel models with fixed effects[a] using symptoms of anxiety as outcome, stratified by sex. [SLOSH=Swedish Longitudinal Occupational Survey of Health, WEHD=Work Environment and Health in Denmark, CI=Confidence interval]

**Workplace bullying to symptoms of anxiety**			**Contemporaneous effects**		**Lagged effects**	
		**N**	**b**	**95% CI**	**b**	**95% CI**
**Women**	**SLOSH**	3281	0.74*	0.18-1.31	-0.05	-0.43-0.32
	**WEHD**	3567	0.47	-0.10-1.04	-0.14	-0.47-0.19
	**Total**	6848	0.61*	0.21-1.01	-0.10	-0.35-0.15
**Men**	**SLOSH**	2187	0.23	-0.45-0.90	-0.03	-0.48-0.42
	**WEHD**	2951	0.89	0.38-1.39*	0.25	-0.04-0.54
	**Total**	5138	0.65*	0.25-1.05	0.17	-0.08-0.41
**Symptoms of anxiety to workplace bullying**						
**Women**	**SLOSH**	3281	-0.00	-0.02-0.01	-0.01*	-0.02-0.00
	**WEHD**	3567	-0.01	-0.03-0.01	-0.02*	-0.03--0.01
	**Total**	6848	-0.01	-0.01-0.00	-0.01*	-0.02--0.01
**Men**	**SLOSH**	2187	0.01	-0.01-0.03	0.00	-0.01-0.01
	**WEHD**	2951	0.03	-0.00-0.07	0.01	-0.00-0.03
	**Total**	5138	0.01	-0.00-0.03	0.01	-0.00-0.01

[a] Models adjusted for job strain (predetermined) and alpha (latent variable representing all time-stable characteristics).
* p<0.05 **p<0.001

Supplementary table S6. Results from attrition analysis of included and excluded participants (who were gainfully employed at time 1) in SLOSH and WEHD[a]. [SLOSH=Swedish Longitudinal Occupational Survey of Health, WEHD=Work Environment and Health in Denmark.]

**SLOSH**			
	**Included**<br>N=5869<br>%	**Excluded**<br>N=7703<br>%	**Statistically significant difference**[b]
**Sex**			**
Female	60.0	54.7	
Male	40.0	45.3	
**Mean age (SD)**	50.3(8.3)	52.8(10.5)	**
**Marital status**			*n.s.*[c]
Married/cohabiting	79.3	80.0	
Single	20.7	20.0	
**Educational attainment**			**
Low	40.3	51.4	
Intermediate	7.9	7.2	
High	51.8	41.4	
**Exposure workplace bullying**			*n.s.*
Yes	7.9	7.3	
No	92.1	92.7	
**Mean anxiety score (SD)**	2.0(2.5)	2.1(2.7)	*
**WEHD**			
	**Included**<br>N=7622<br>%	**Excluded**<br>N=18077<br>%	**Statistically significant difference**[b]
**Sex**			*n.s*
Female	55.2	54.1	
Male	44.8	45.9	
**Mean age (SD)**	46.6	44.6	**
**Marital status**			**
Married/cohabiting	80.3	76.9	

Single	19.7	23.1	
**Educational attainment**			**
Low	53.9	61.5	
Intermediate	32.8	27.8	
High	13.4	10.7	
**Exposure workplace bullying**			*n.s.*
Yes	12.2	12.5	
No	87.8	87.5	
**Mean anxiety score (SD)**	1.7(2.5)	1.8(2.4)	*

[a] All items measured at year of first wave included in study (SLOSH: 2016, WEHD: 2012).
[b] According to chi-square test of difference (categorical variables) or t-test (continuous variables)
[c] n.s.= not significant
**p<0.001
*p<0.05
